# A unique, interactive and web-based pediatric rheumatology teaching module: residents’ perceptions

**DOI:** 10.1186/1546-0096-11-22

**Published:** 2013-05-27

**Authors:** Michelle Batthish, Ereny Bassilious, Rayfel Schneider, Brian M Feldman, Avi Hyman, Shirley ML Tse

**Affiliations:** 1 Division of Rheumatology, Department of Pediatrics, McMaster Children’s Hospital, McMaster University, 1280 Main Street West, Hamilton, Ontario, L8S 4L8, Canada; 2 Division of Endocrinology, Department of Pediatrics, McMaster Children’s Hospital, McMaster University, 1280 Main Street West, Hamilton, Ontario, L8S 4L8, Canada; 3 Division of Rheumatology, Department of Pediatrics, The Hospital for Sick Children, University of Toronto, 555 University Avenue, Toronto, Ontario, M5G 1X8, Canada; 4 Institute of Health Policy Management & Evaluation and The Dalla Lana School of Public Health, University of Toronto, 155 College Street, Toronto, Ontario, M5T 3M6, Canada; 5 Academic and Collaborative Technology, University of Toronto, 130 St. George Street, Toronto, Ontario, M5S 3H1, Canada

**Keywords:** Web-based learning, Pediatric residents

## Abstract

**Background:**

The limited availability of pediatric rheumatologists for teaching in pediatric residency programs negatively impacts resident education about rheumatology. At present, there are no educational websites available for trainees to learn about pediatric rheumatology. We are planning to develop an interactive web-based teaching module to improve resident learning about rheumatology (“POINTER”: Pediatric Online INteractive TEaching in Rheumatology). The aim of this study was to perform a needs assessment of pediatric residents who will be using POINTER.

**Methods:**

Pediatric residents (n = 60) at The Hospital for Sick Children were emailed an online survey. This was designed to assess prior use of online teaching modules, the utility of an online teaching module for rheumatology and which technologies should be included on such a site.

**Results:**

Forty-seven residents participated in the survey (78.3% response rate). Ninety-one percent of the respondents thought that an interactive teaching website would enhance their learning and should include case-based teaching modules. Several web-based technologies were felt to be important for inclusion on the teaching modules. These included graphics and animation (86.4%), interactivity (93.2%), pictures (100%), live digital videos (88.9%) and links to articles and research (88.6%).

**Conclusions:**

An interactive web-based rheumatology teaching module would be well utilized by pediatric residents. Residents showed preference for case-based teaching modules as well as multimedia modalities for learning a detailed musculoskeletal examination.

## Background

There exist widespread deficiencies in pediatric musculoskeletal (MSK) training. Evidence shows poor performance of pediatric MSK assessment in clinical pediatric practice [1], and trainees in primary care report that training in pediatric MSK assessments is inadequate [2]. Furthermore, exposure to pediatric rheumatology training is limited to centers with pediatric rheumatologists [3] and formal instruction in pediatric MSK assessment is lacking within pediatric residency programs [4]. In Canada, there are only three pediatric rheumatology training centers. Therefore, a large number of pediatric residents nationwide do not receive teaching about pediatric musculoskeletal conditions from expert subspecialists and may lack exposure to patients with these conditions.

The internet and web-based technologies are playing an increasingly larger role in medical education. To date, there exist few computer-assisted learning programs for rheumatology trainees. The Interactive Rheumatology Tutor, initially developed in 1997, is aimed at undergraduates, general medical and rheumatology post-graduate trainees and general practitioners [5]. It contains many images and includes video and audio clips. The American College of Rheumatology (ACR) has both an online tutorial system aimed at medical students and primary care physicians as well as a case-based curriculum on CD-ROM [6]. Many medical schools and university websites also contain rheumatologic educational content. At present, there are no educational websites available for trainees to learn specifically about pediatric rheumatology.

Computer-assisted learning offers many advantages over conventional teaching methods. There is the potential to utilize multi-media applications with interactivity. It also allows users to learn at their own pace as well as at their own convenience. The creation of an interactive learning environment has the potential to transform learning away from basic acquisition of facts (i.e. printed materials, didactic lectures, basic online repositories of information), to actively acquiring and applying knowledge, skills and approaches necessary in becoming a competent and efficient clinician. An innovative, online learning environment can have a substantial impact on student learning and problem solving [7]. Technical advantages of web-based learning include universal accessibility, ease in update content, and hyperlink functions that permit cross-referencing to other resources [8].

We are planning to develop an innovative, interactive web-based teaching module as a means to improve core pediatric resident learning and problem solving about pediatric rheumatology (“POINTER”: Pediatric Online INteractive TEaching in Rheumatology). Prior to developing any medical education curriculum, a needs assessment for the targeted learners is essential [9]. The aim of the present study was to perform a needs assessment of core pediatric residents who will potentially be using POINTER to further understand their acceptability and requirements of this new web-based educational tool.

## Methods

Pediatric residents (Year 1 to Year 4, n = 60) at The Hospital for Sick Children (SickKids), Toronto, Canada were emailed a link to an online survey for completion via SurveyMonkey. SurveyMonkey was chosen for its feasibility to administer the survey in accessing all the residents, protecting the anonymity of the responders and ensuring their participation was voluntary. The survey comprised of 9 close-ended questions and 3 open-ended questions (Additional file 1: Table S1). Residents were informed that the Division of Rheumatology at SickKids was in the process of developing an online interactive web-based teaching module for residents to learn about pediatric rheumatology (POINTER). They were asked to complete a voluntary needs assessment survey to determine their interest in and potential usage of this module and to identify specific items or features that residents felt were important and should be included in the development of POINTER.

This study was approved by the SickKids research ethics board.

## Results

Forty-seven pediatric residents participated in the survey (78.3% response rate). Seventeen (36.2%) were in their first year of training, 11 (23.4%) were in their second year, 14 (29.8%) were in their third year and the remaining 5 (10.6%) were in their final year of training. Twenty-one of the 47 residents (44.7%) had completed a subspecialty rotation in rheumatology.

Almost all the respondents (91.5%) thought that an interactive teaching web site would enhance their learning about childhood arthritis and rheumatic diseases. Half of the residents (51.1%) had accessed other teaching web sites to learn about pediatric diseases. The most frequent online tool reported to be utilized amongst the residents was the Computer assisted Learning in Pediatrics Program (CLIPP). Thirty-six respondents (76.6%) would definitively use a web site to learn about rheumatic diseases, while 11 (23.4%) would consider using it.

**Figure 1:**
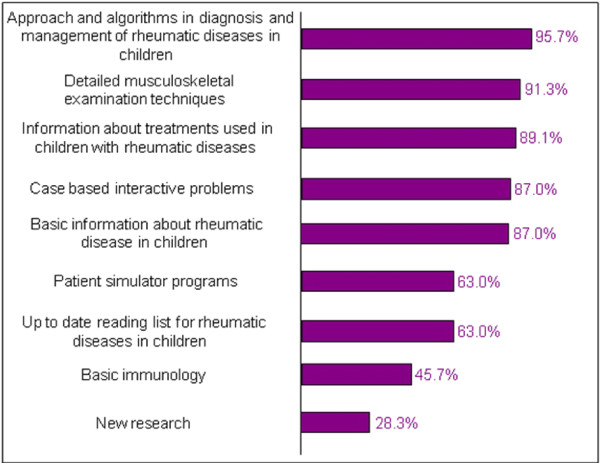
Percentage of residents who would like to see the following content included on a rheumatology teaching web site.

When asked about what type of information should be included on a rheumatology web site, most respondents suggested approaches and algorithms for the diagnosis and management of rheumatic diseases (95.7%) as well as information about treatments used for children with rheumatic diseases (89.1%) (Figure 1).

**Figure 2:**
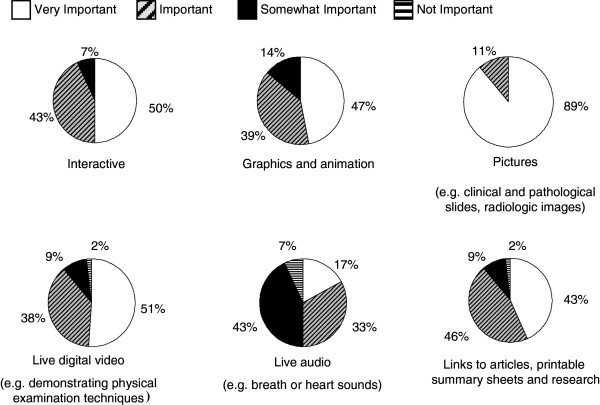
**Percentage of residents who felt that the following web**-**based technologies should be included on a rheumatology teaching website to make it more effective.**

Demonstration of detailed musculoskeletal examination techniques was considered a very important component to the teaching web site (91.3%) while an up-to-date reading list was felt to be important but less so (63.0%). Case-based interactive problems were supported by more residents than patient simulation programs (87.0% vs 63.0%). Several web-based technologies were felt to be important or very important for inclusion in the online teaching modules. These included graphics and animation, interactivity, pictures of disease manifestations, live digital videos and links to articles and research (Figure 2). General comments expressed by the residents included support for the initiative and that the development of the online tool was indeed necessary and timely to assist in improving their learning in pediatric rheumatology.

## Discussion

Based on the current needs assessment, an interactive web-based pediatric rheumatology teaching module would be feasible and is likely to be well utilized by pediatric residents. Almost all the respondents thought that an interactive teaching website would enhance their learning about childhood arthritis and rheumatic diseases. Residents showed a preference for several multimedia modalities, including graphics, animation and live digital video. These features will be incorporated into our POINTER website to encourage ongoing use. Although a patient simulation program was supported by fewer residents, this feature will be combined with algorithmic approaches in our website to enhance resident learning. We plan to formally evaluate this feature in the next phase of the development of POINTER to ensure that residents are using and are satisfied with this modality. Similarly, up-to-date reading lists will be integrated into the case-based interactive simulations to promote their use.

Computer-assisted learning is being used increasingly in medical schools and residency training. It has been shown to be equivalent to traditional teaching but is associated with greater learning efficiency, problem solving abilities and satisfaction [10-12]. One online patient simulator and tutorial did not improve the quantity of material learned but it did improve learning efficiency [13]. When comparing a web-based tutorial to printed materials for self-study, Bell et al found that a teaching website improved residents’ learning efficiency and satisfaction more than self-study [10]. Undergraduate students exposed to a web-based learning program scored significantly better on a final examination and expressed a positive attitude to the new learning environment in a satisfaction survey [12]. In a review of the medical web-based learning literature, these types of programs were not found to be superior to traditional methods in terms of gains in learning or learners’ satisfaction [14]. Similarly, in a recent meta-analysis, while internet-based learning was associated with large positive effects compared with no intervention, when these effects were compared to non-internet instructional methods, they were inconsistent and generally small, suggesting effectiveness similar to traditional methods [15]. It remains to be determined if computer-assisted learning is associated with greater learning efficiency and whether or not this form of learning is more acceptable and accessible to learners. From our survey, the high acceptability for the pediatric rheumatology online tool may be attributed to having a repository of disease specific curriculum in a single site that is easily accessible and allows for individualized learning.

The development of an online teaching website must incorporate several features to optimize learning efficiency. The modules should include an interactive component with ongoing feedback. Students highly appreciate feedback models incorporated in an interactive computer program [16]. In addition, the inclusion of multi-media, such as digital images and video, is also an essential component in any online teaching module. Previous studies have shown that computer-assisted learning can be successfully implemented with positive user satisfaction when such features are included [16-18]. In a recent meta-analysis of internet-based learning for health professions education, interactivity, repetition and feedback seem to be associated with improved learning outcomes [19]. From the survey, our residents have listed similar desired features that are likely aligned with optimizing their learning efficiency and engagement. As the diagnosis of various rheumatic diseases is often dependent on the physical exam, it is not surprisingly that our learners rated the inclusion of musculoskeletal examination techniques and multi-media (pictures and video) to be important. A single-exposure to a self-study module may be insufficient to achieve long-term retention. Although computer-assisted learning can increase learning efficiency, online modules must be created to encourage ongoing use (for example, by creating multiple case scenarios or one case scenario with multiple potential outcomes) to improve long-term retention. This unique feature will be incorporated in the POINTER website.

One of the limitations in our study is the relatively small sample of residents surveyed from a single pediatric institution. They were at different levels of training and experience in pediatric rheumatology. We were not able to cross-tabulate the data to separate these responses, including those residents who had already completed a rotation in pediatric rheumatology prior to the survey. There was also a likely selection bias since our survey was administered online and this would have selected residents who are more likely to use computer-assisted learning modules. However, we had a very high response rate. It may be that we have slightly overestimated the enthusiasm with which postgraduate pediatric trainees will embrace this style of learning in pediatric rheumatology. One final limitation is social desirability bias in that the residents are very likely to endorse any added learning no matter what form suggested. Whether or not they will ultimately use it with good effect on knowledge and attitude outcomes is downstream and will need to be further explored. However, once POINTER is developed, we will be able to better assess resident use and satisfaction with this teaching module.

## Conclusions

In conclusion, an interactive web-based pediatric rheumatology teaching module would be accessible and is likely to be well utilized by pediatric residents. At present, there are no other educational websites available for pediatric rheumatology that incorporates an innovative online learning environment (combining interactive case scenarios or patient simulators). We are in the process of developing an online interactive web-based teaching module for residents to learn about pediatric rheumatology (POINTER). Following development of POINTER, we will evaluate the feasibility, effectiveness and satisfaction of this module.

## Competing interests

The authors report no competing interests.

## Authors’ contributions

MB collected and analyzed the survey data and drafted the manuscript. EB contributed to the interpretation of survey data and read and approved the final manuscript. RS contributed to the interpretation of survey data and read and approved the final manuscript. BMF contributed to the interpretation of survey data and read and approved the final manuscript. AH participated in the design of the website and read and approved the final manuscript. SMLT conceived of the study, participated in its design and coordination and read and approved the final manuscript.

## Supplementary Material

Additional file 1: Table S1 Online needs assessment survey given to pediatric residents. Click here for file
