# Supplementary material for: A unique, interactive and web-based pediatric rheumatology teaching module: residents’ perceptions
Source: Pediatr Rheumatol Online J. 2013 May 27;11:22. doi: 10.1186/1546-0096-11-22 (PMC3679723; doi:10.1186/1546-0096-11-22)
Supplement: Additional file 1: Table S1 — Online needs assessment survey given to pediatric residents. [file 1546-0096-11-22-S1.doc]

**Table 1**: Online needs assessment survey given to pediatric residents.

| **SURVEY: Rheumatology WEB Based Teaching Module**  1. My current level of training as a resident is:  □ PGY-1  □ PGY-2  □ PGY-3  □ PGY-4  □ PGY-5  □ Medical student  □ I am not a core pediatric resident. Current training level is_______________________.  2. I have completed or am completing a subspecialty rotation in rheumatology.   - Yes □ No   3. Do you have access to a computer? (Check all that apply)  □ At home  □ At work  □ At the library  □ No access to a computer  4. Do you have access to the Internet? (Check all that apply)  □ At home  □ At work  □ At the library  □ No access to the internet  5. What type of internet access do you use mostly for education?  □ High Speed (cable or DSL)  □ Dial up  □ No access to the internet  6. Would you find it helpful for the Division of Rheumatology to develop an interactive teaching web site to learn about childhood arthritis and rheumatic diseases?  □ Yes □ No □ Not sure  7. Do you think you would use a web site to learn about rheumatic diseases?  □ Yes □ Yes somewhat □ Never □ Not sure  8. Have accessed any teaching web sites to learn about pediatric diseases  □ Yes □ No □ Not sure  If yes, please give example(s) of a teaching website you found helpful, educational or effective  If yes, did any of the teaching web sites have an interactive component? For example, working through a patient scenario and not simply providing reading material.  □ Yes □ No □ Not sure  9. What information would you like to see included on a rheumatology teaching web site for residents?  □ Basic information about rheumatic diseases in children  □ Approach and algorithms in diagnosis and management of rheumatic diseases in children  □ Case based interactive problems  □ Patient simulator programs  □ Information about treatments used in children with rheumatic diseases  □ Detailed musculoskeletal examination techniques  □ Up to date reading list for rheumatic diseases in children  □ New research  □ Basic immunology  □ Other. If you selected other, can you give us an ideal of the kinds of information or activities you think should be included on this web site?  ____________________________________________________________________________________________________________________________________________________________________________________________________________________________________________________________________________________________________  10. Which web based technologies do you feel should be included on the web site to make it more effective?  □ interactive  □ graphics and animation  □ pictures (clinical and pathological slides, radiologic images)  □ live digital video (e.g. demonstrating physical examination techniques or procedures such as joint aspiration)  □ live audio (breath or heart sounds)  □ links to articles, printable summary sheets, research  11. Do you feel that this rheumatology web site will help you with:  □ diagnosis and management of patients in your clinical practice  □ acquiring new knowledge about children with rheumatic diseases  □ preparing and passing your pediatric license exams  □ not helpful and needed at this time  12. If you have any further comments or suggestions about this initiative, please share them with us?  _________________________________________________________________________________________________________________________________________________________________________________________________________________________________________­­­­­­­­­­­­_______________________________________________________________________ |
| --- |
